# Adjunctive treatment of chronic migraine using an oral dental device: overview and results of a randomized placebo-controlled crossover study

**DOI:** 10.1186/s12883-022-02591-8

**Published:** 2022-03-04

**Authors:** Andrew M. Blumenfeld, James P. Boyd

**Affiliations:** The Los Angeles and San Diego Headache Centers, Los Angeles, CA USA

**Keywords:** Clenching, Migraine, Nociceptive, NTI Splint, HIT-6 score

## Abstract

**Objective:**

To assess the nocioceptive input of habitual nocturnal jaw clenching that acts as a contributing factor in migraine pathogenesis.

**Background:**

Habitual nocturnal jaw clenching has been implicated as a trigger, particularly in those whose headaches are present upon waking or shortly thereafter. Nocturnal EMG studies of patients diagnosed with migraine show nearly twice the temporalis clenching EMG levels and double the bite force as matched asymptomatic controls, leading to the speculation that parafunctional clenching activity may have some role in headache pathogenesis. The NTI (Nociceptive Trigeminal Inhibition) oral device is a dental splint designed to reduce nocturnal jaw clenching intensity and is FDA approved for the prevention of medically diagnosed migraine pain based on open label studies. There are no prior placebo-controlled trials to assess the migraine prevention efficacy of the NTI splint. This is the first placebo-controlled cross-over study to assess the efficacy of the NTI splint in patients with Chronic Migraine.

**Method:**

A placebo controlled, single-blinded cross-over study was done with IRB oversight assessing the efficacy of the NTI splint compared to placebo using the change in the HIT-6 score as the outcome measure.

**Results:**

68% of refractory chronic migraine sufferers using the NTI as measured by sequential HIT 6 scores had at least a one-category improvement (severe to substantial, or substantial to some, or some to none) compared to 12% when using a placebo device. 36% of subjects using the NTI device reported a two-category improvement in their HIT-6 score, compared to 0% when using placebo.

**Conclusion:**

The improvement in HIT-6 scores produced by the NTI device, suggests that patients with Chronic Migraine may have intense nocturnal jaw clenching as a contributing factor to their headache related disability. An NTI device is one method of assessing whether jaw-clenching is a contributing factor to ongoing migraine.

**Trial registration:**

Current Controlled Trials NCT04871581. 04/05/2021. Retrospectively registered.

## Introduction

Chronic Migraine is one of the most disabling conditions and persons with this condition have greater disability and lower quality of life than persons with episodic migraine and they often overuse headache medications [1–4].

Many patients with Chronic Migraine are refractory to multiple preventive medications and others are reluctant to use pharmacotherapeutic treatments due to their systemic side effects [5]. In addition, many of these patients are of child-bearing age and have concerns that these medications may produce teratogenic effects or miscarriages if they become pregnant.

Migraine is an episodic disorder of neuronal dysfunction involving the trigemino-vascular system. As the condition becomes chronic, sensitization occurs of the central sensory pathways [6], leading to.

changes in pain and sensory input processing [7]. Pain and sensory input to the trigeminal sensory nucleus is not limited to the first and second divisions of the trigeminal nerve. Many Chronic Migraine patients have parafunctional clenching activity and features of TMJ dysfunction possibly due to trigeminal motor hyperactivity and dysfunction of the third division of the trigeminal nerve [8]. The role of habitual nocturnal jaw clenching as a contributing factor in migraine pathogenesis has not been established but has been implicated as a trigger [9], particularly in those whose headaches are present upon waking or shortly thereafter [10].

Jaw-clenching and bruxism can be nociceptive in nature. These activities occur in association with arousals from deep sleep and occasionally REM sleep [11]. Deep sleep predominates in the early portion of the sleep period while REM sleep occurs more toward the end of the sleep period. The timing of this nociceptive input may be a trigger for headache on waking.

Daily resting ambulatory EMG levels of the temporalis (the primary jaw clenching muscle) in persons with migraine is not significantly different than matched asymptomatic controls [12, 13].

Nocturnal EMG studies of patients diagnosed with migraine show nearly twice the temporalis clenching EMG levels and double the bite force as matched asymptomatic controls, leading to the speculation that parafunctional clenching activity may have some role in headache pathogenesis [14]. Traditional mouthpieces (or “splints”) used to treat temporomandibular disorders are designed to reduce strain and load on the TM joint while the patient is clenching on the splint. This is accomplished by providing an ideal bi-lateral posterior clenching surface on the splint which immobilizes and stabilizes the joint. However, this occlusal design provides an ideal clenching surface for the molars and therefore cannot curtail potential temporalis jaw-clenching intensity, and may allow for jaw-clenching to intensify [15, 16] which may perpetuate migraine symptoms, resulting in the conclusion that “splints” are ineffective.

When using a splint to treat a population of patients who have headache attributed to a TM disorder, approximately 1/3 discontinue use due to an increase of symptoms, 1/3 will report no effect, while only 1/3 will improve [17, 18].

Intra-oral splints have traditionally been sub-divided into two broad categories: the full coverage splints, which cover an entire dental arch; and the partial coverage splints, which cover either the posterior molar segments, or the anterior segment (from canine to canine tooth). By providing for molar and/or canine contact through their long axis, both designs allow for ungoverned clenching intensity. The Nociceptive Trigeminal Inhibition Tension Suppression System (NTI) is a partial coverage device that minimizes clenching intensity and the subsequent afferent nociceptive activity by providing for only incisor’s edges occluding contact on the splint (while ensuring canine and posterior molar separation). The NTI is a semi-custom intraoral mouthpiece approved by the FDA in 2001 for the prophylactic treatment of medically diagnosed migraine pain through reduction of trigeminally-innervated muscular activity [19].

## Methods

A placebo controlled cross-over pilot study was done with IRB oversight to assess the efficacy of the NTI splint using the change in the HIT-6 score as the primary outcome measure. A power analysis was not done, as this pilot study’s results will indicate the signal if a larger study is warranted. The HIT-6 score was selected as the outcome measure, as the goal was to focus on improving disability, as in chronic migraine, changes in headache days may not be as helpful as improving disability [20]. This was a single blind study and participants were treated with both a placebo device and an NTI device. The participants were not aware of which device was FDA approved, while practitioners were familiar with the NTI.

Thirty consecutive refractory Chronic Migraine (CM) patients managed at a Neurology Headache Center, were referred to a dental practice to be considered for the study. 25 consented and completed a baseline Headache Impact Test (HIT-6) questionnaire [21, 22]. The HIT-6 measures both the severity of pain and the adverse impact that headache has on the quality of the sufferer’s life.

For this study a positive result was a one-level shift to a lesser HIT-6 category negative impact after use of the oral device:

- Little or no impact;
- Some impact with considerable pain;
- Substantial impact with severe pain;
- Severe impact with disabling pain**.**

**Fig. 1:**
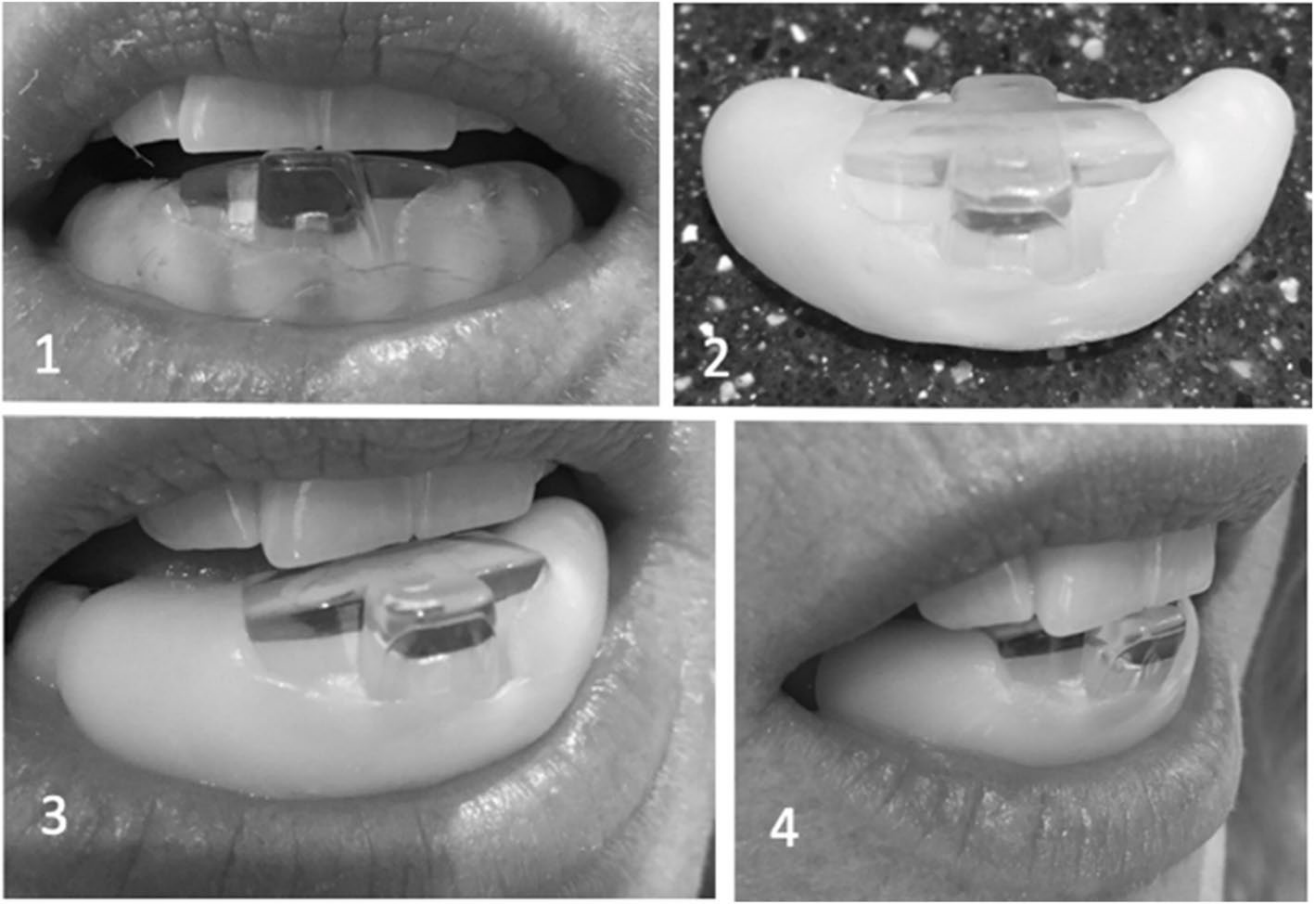
NTI device. 1) Initial closure. 2) Lower device 3) protrusive position. 4) retrusive position. The device must be modified to ensure incisor edge contact in the extremes of each position

**Fig. 2:**
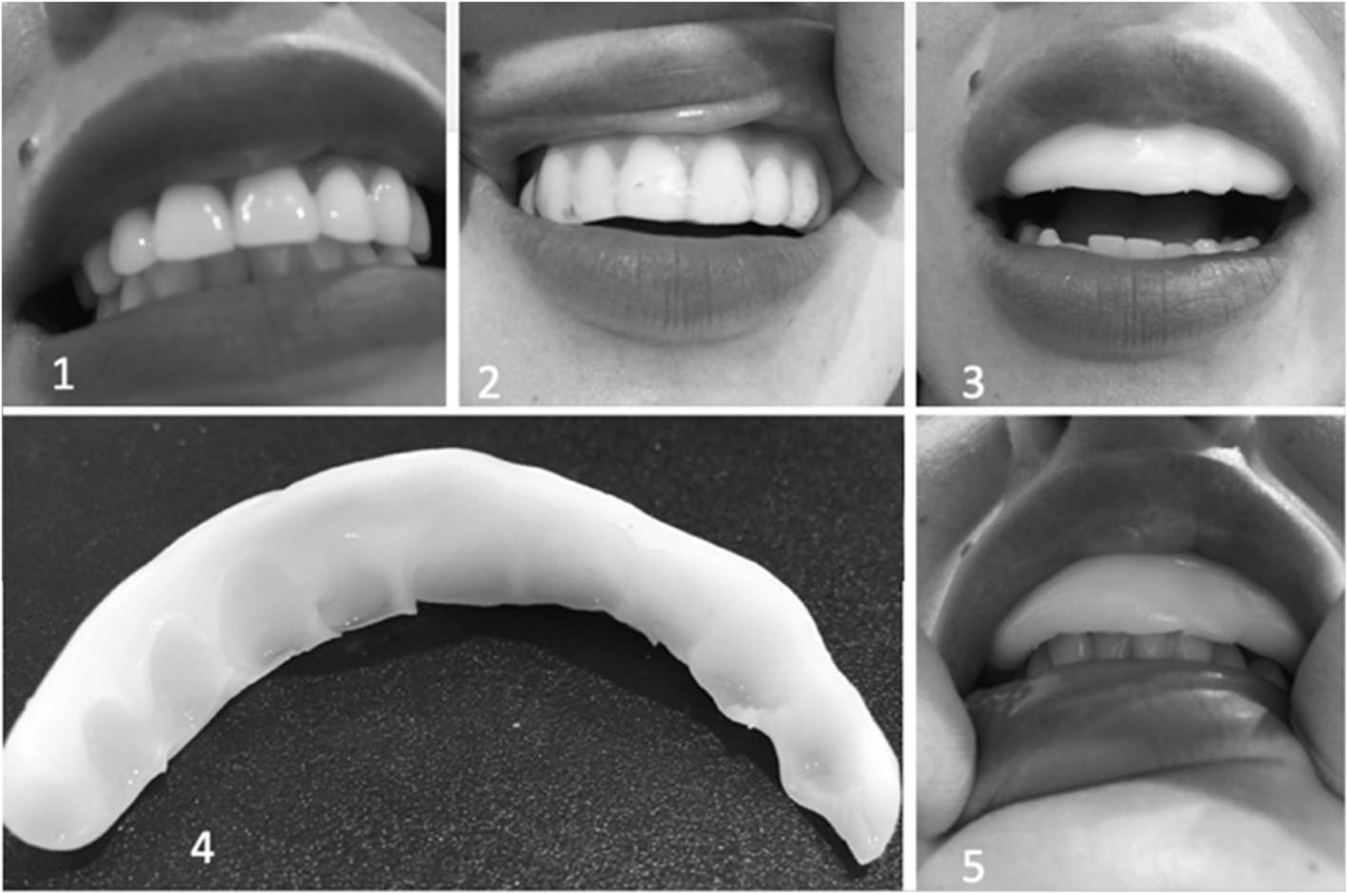
Placebo device. 1. No device. 2. Warmed thermoplastic in place 3. Cooled thermoplastic. 4. Final placebo device. 5. Placebo in place

The subjects were consented and informed that this study was designed to compare the efficacy of migraine prevention, if any, provided by two different intraoral devices that were worn during sleep that were positioned and retained in the anterior region of the mouth. No other descriptions or details of the devices to be used were discussed with the subjects. Each subject was then treated with either an NTI device (Fig. 1) or a placebo device (Fig. 2).

The placebo device was custom fabricated on each subject by adapting a thermoplastic putty over the gingival surface within the maxillary labial sulcus spanning from maxillary canine to canine. The placebo intraoral device had no impact on the subject’s dental occlusion and no influence on nocturnal jaw muscle activity as it did not limit contact of dentition important for clenching activity.

This study used a pre-fabricated NTI device lined with the same thermoplastic used for the creation of the placebo.

**Fig. 3:**
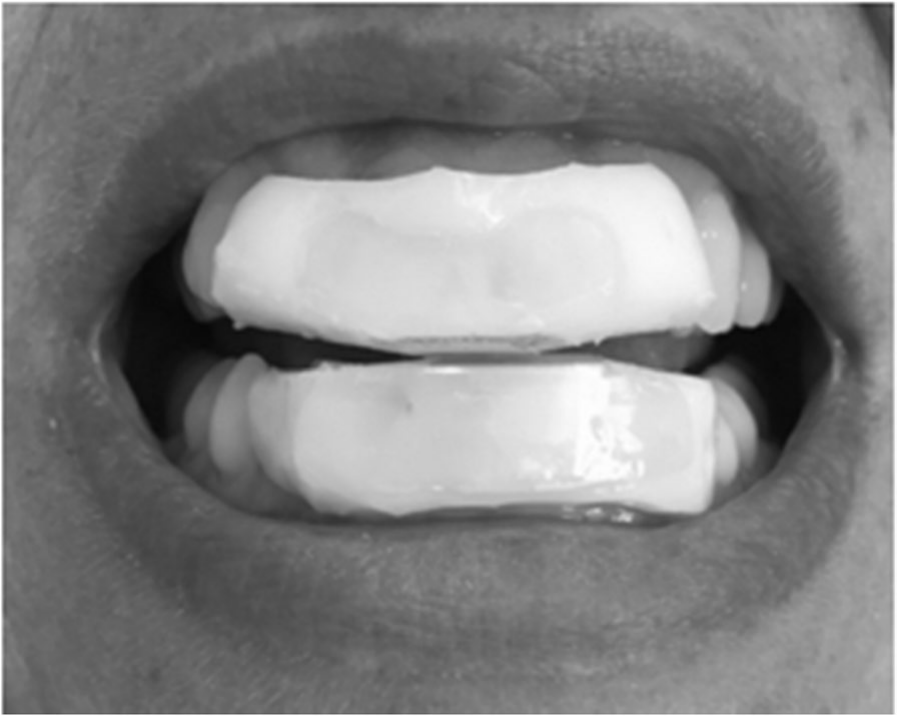
Opposing NTI devices to provide for minimal lateral resistance. The greater the surface area of contact, the more intense the clenching activity can be, therefore, the contacting surface area should be minimized

As per the manufacturer’s recommended protocol, each NTI device was customized to the subject to minimize temporalis contraction intensity by providing for an anterior mid-point contact with minimal vertical dimension of occluding (VDO). In those subjects whose incisors opposing the NTI device were irregularly aligned and/or whose incisal edges provided an irregular and non-continuous surface, an additional device was adapted over the opposing incisor’s edges to provide a smooth and continuous surface (Fig. 3).

**Fig. 4:**
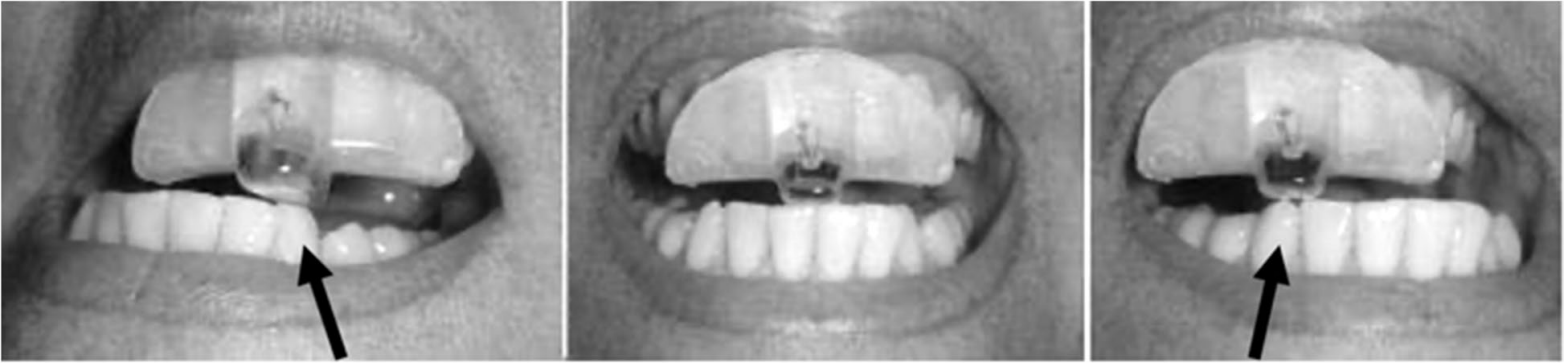
Improper NTI Protocol. Arrows indicate canine contact on the NTI device when the patient’s jaw shifts laterally. Canine contact allows for intense trigeminal motor hyperactivity (clenching), creating considerable strain to the contralateral temporomandibular joint, potentially producing excessive nociception

**Fig. 5:**
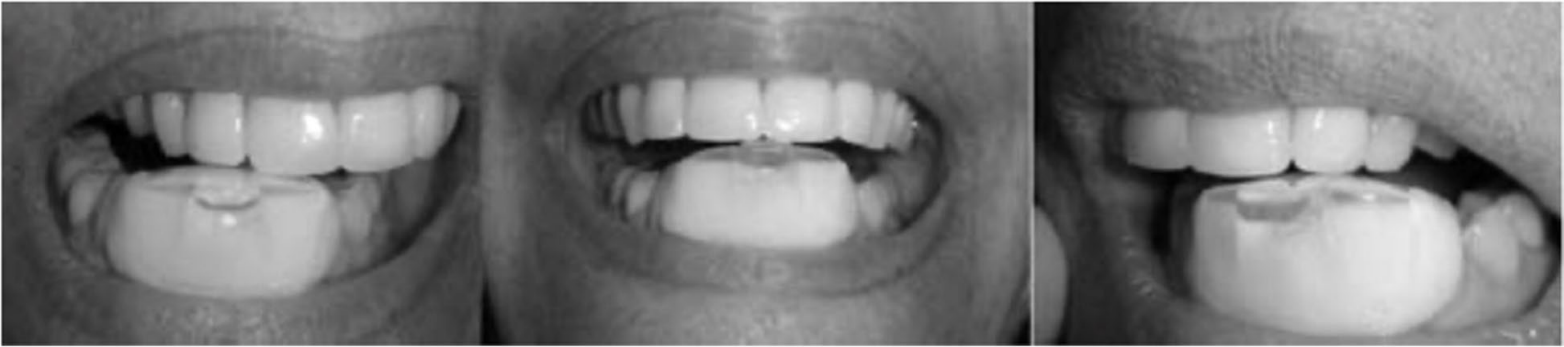
Corrected NTI protocol. By maintaining incisor edge contact and preventing canine or posterior tooth contact during the parafunctional acts, the properly-aligned NTI keeps both the muscle contraction intensity and strain on the distracted temporomandibular joint to a minimum

The NTI device needs to be manufactured by a dentist knowledgeable in making this correctly (http://www.nti-tss.com/OVERSIGHTS.html). For example, in order for the activity of the trigeminally-innervated temporalis muscle to achieve pathologic intensity, either a canine or posterior molar must come into contact with either another tooth or the device itself [23]. The NTI device therapy fails if either a canine or posterior tooth chronically comes into contact with another tooth (or the device) during nocturnal parafunctional hyperactivity. Figure 4 illustrates an NTI device that had been improperly provided. Figure 5 demonstrates an NTI device that has been properly provided.

**Fig. 6:**
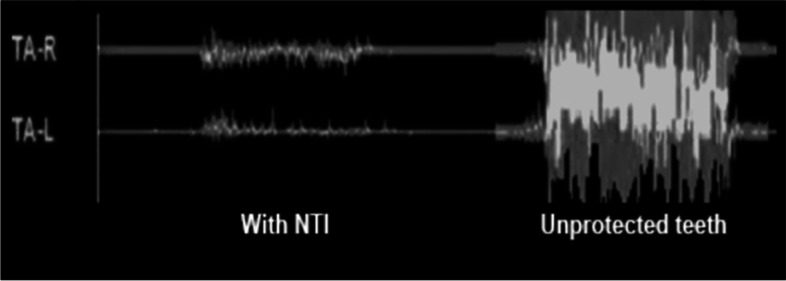
Temporalis EMG of Maximum Clenching Intensity over the anterior left and right temporalis muscle (TA-L and TA-R)

The EMG recordings shown in Fig. 6 demonstrate the effect that the well-designed device has on trigeminal motor maximum clenching activity. When used nightly over a six-month period, maximum temporalis motor activity as measured by EMG is reduced by over 50%  [24]^.^

Subjects were randomized 1:1 to either 30 days of use of the NTI splint or 30 days of use of the placebo splint. After 30 days of nightly use, subjects completed another HIT-6 and were then switched to the alternate device. No mention of the subject’s previous or current HIT-6 score, or the nature of the devices being delivered, were discussed or mentioned. After another 30 days of nightly use, subjects completed a final HIT-6.

Limitations of the study: Single blind leading to potential bias.

## Results

**Fig. 7:**
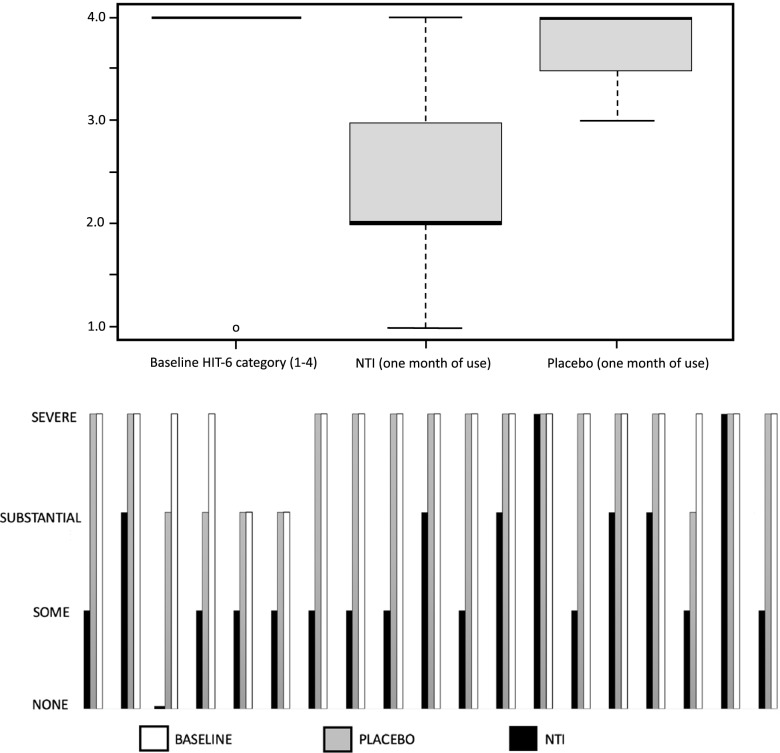
Comparison of effect of NTI vs Placebo on degree of negative impact of Chronic Migraine on individual subjects
- 36% (9 of 25) of subjects using the NTI device reported a two-category improvement in their HIT-6 score, compared 0% when using placebo (Figs. 8 and 9).

- 19 of the 25 subjects completed the 2-month trial. (the 6 dropouts either failed to return for check-ins, or did not comply with the protocol)
- Initial baseline average HIT-6 of all subjects: Category 4: “Severe impact, disabling pain”
- 68% of subjects (17 of 25) using the NTI device had at least a one-category improvement in their HIT-6 scores compared to 12% (3 of 25) when using placebo (Fig. 7).

**Fig. 8:**
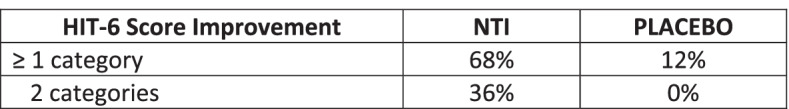
Reduction in categories of negative impact

**Fig. 9:**
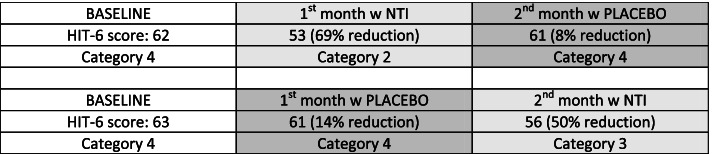
Crossover comparisons of the 19 subjects who completed the trial, changes in HIT-6 scores for each group. (a HIT-6 score of ≤ 49 equates to no impact on the subject’s life (percent changes are therefore relative to a score of 49)
- There were no adverse effects reported by any of the subjects

Statistical analysis in reduction in categories of negative impact.

**Table:** 

HIT-6 Score Improvement	NTI	Placebo
≥ 1 category	14/19 = 74%	3/19 = 16%
2 categories	9/19 = 47%	0%

The Fisher test was implemented to see if there was a statistically significant difference in HIT-6 Score Improvement (rather than a Chi-Square, as one table`s cell contained less than 5 observations and the sample size was less than required 40, which are assumptions of the Chi-square test).

The Fisher test revealed (*p* = 0.00125), that there is a statistically significant difference between NTI and Placebo improvement.

The non-parametric Friedman’s test, a non-parametric test for finding differences in treatments across multiple attempts, revealed that there was significant difference between baseline HIT-6 scores, NTI and Placebo values (X(2) = 32.4, *p* <  < 0.001).

## Discussion

Migraine is a disease that involves overactivity in the trigeminal-occipital-cervical complex of the nerves. These afferents feed into the trigeminal nucleus caudalis. Decreasing peripheral sensitization with modalities such as the NTI splint protocol which reduces chronic nociceptive input can help to decrease central sensitization, which is a hallmark of Chronic Migraine.

The purposeless nocturnal parafunction of jaw clenching, with or without forcible excursive movements (grinding), can produce abundant nociceptive input to the sensory nucleus, with the intensity of contraction of the elevator muscles dictating the degree of nociception. As elevator contraction intensity increases, so does the pressure and strain on the teeth, the periodontal ligaments and temporomandibular joints, while the antagonist muscles (lateral pterygoids) strain to depress the mandible and separate the teeth. During unilateral posterior clenching in excursive mandibular positions, considerable strain is endured by the distracted contralateral joint.

When elevator intensity (degree of jaw clenching) is minimized, the force of contact on the teeth and resulting nociceptive input is also minimal, thereby presenting the lateral pterygoids with less resistance to their activity which minimizes the potential direct strain and load on the TM joints.

Therefore, the therapeutic design of a nocturnal intraoral device would be to provide nocturnal full-time posterior disclusion with incisor edge contact (“incisal guidance” [25]) to minimize the influence of chronic nocturnal nociception on the triggering of a headache by ensuring two criteria:

1. to minimize jaw-clenching intensity (primarily the temporalis), by providing for incisor edge contact, only;
2. to minimize TM joint strain and load [26] by minimizing the resistance encountered by the lateral pterygoids (via the provision of smooth articulated surfaces) and the degree of condylar rotation during the clenching events by minimizing the vertical dimension of occluding (VDO).

**Fig. 10:**
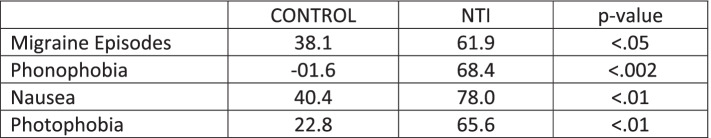
Percent reduction of listed symptoms during second month of NTI use compared to Control Device in the initial FDA trial study

**Fig. 11:**
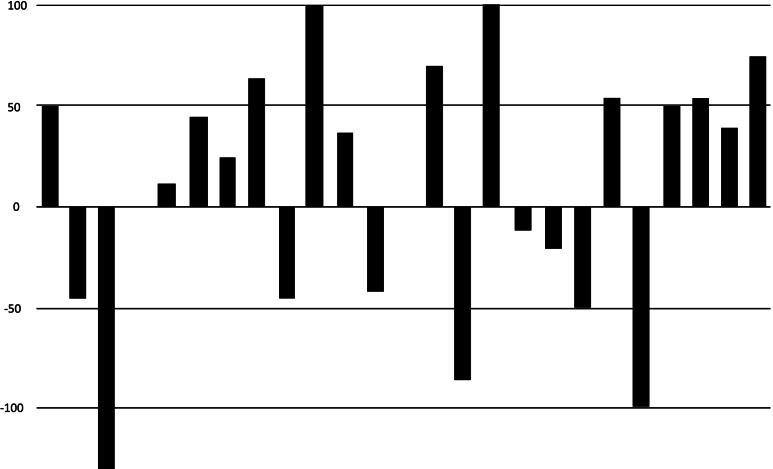
Percent reduction of migraine events of individual full-coverage control splint users (Elkhart site) during second month of use in the initial FDA trial study

For the FDA approval study [27, 28] the NTI was compared with a traditional dental splint as a control device [29], for the prophylactic treatment of migraine pain. During the second month of use, overall, the control device performed as expected, while the NTI was nearly twice as effective (Figs. 10 and 11).

**Fig. 12:**
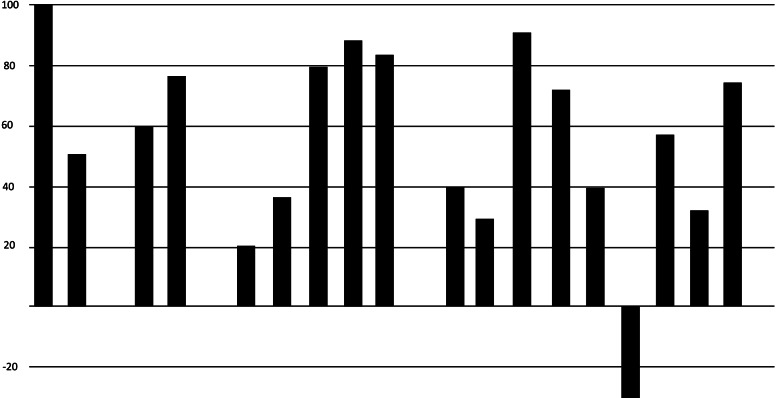
Percent reduction of migraine events for individual NTI users (Elkhart site) during second month of use in the initial FDA trial study

The outcomes for the control splint, nearly as many subjects reporting considerable worsening of symptoms (shown as a negative percent reduction) as those reporting improvements (Fig. 12).

In contrast, 82% of subjects using the NTI-TSS reported a mean 77% reduction in migraine events, with less than 5% reporting an increase in symptoms.

To date, the NTI has yet to become a commonly used adjunctive preventive treatment. The reasons for this may include prior experiences of patients with negative outcome from traditional dental splints, and a lack of knowledge by providers of clenching pathophysiology and this alternative option.

## Conclusion

The improvement produced by the NTI device (Figs. 7, 8 and 9) suggests that Chronic Migraine patients may have nocturnal jaw clenching as a contributing factor, and this may be undiagnosed by health care providers. The production of nociceptive input by the affected teeth, bone, TMJs and muscles to the trigeminal sensory nucleus produced by nocturnal jaw clenching should be considered as a potential perpetuating and confounding co-factor of Chronic Migraine. An NTI device should be considered as a method of assessing whether jaw-clenching is a contributing factor to ongoing migraine.

### Disclosures

NTI devices were provided by ChairsideSplintStore.com, the exclusive distributor of the NTI devices.

Dr. Blumenfeld has acted as a consultant for National Dentex Labs, a provider of NTI devices.

## Data Availability

The datasets used and/or analyzed during the current study are available from the corresponding author on reasonable request.

## Abbreviation

NTI: Nociceptive Trigeminal Inhibition
